# More than my appearance: a pilot evaluation of the expand your Horizon Online functionality-based writing programme for adults with visible differences

**DOI:** 10.1080/21642850.2024.2349004

**Published:** 2024-05-09

**Authors:** Ella Guest, Emma Halliwell, Abbi Mathews, Jessica Alleva, Diana Harcourt

**Affiliations:** a Centre for Appearance Research, University of the West of England (UWE), Bristol, UK; b Department of Clinical Psychological Science, Maastricht University, Maastricht, The Netherlands

**Keywords:** Body functionality, positive body image, writing intervention, visible difference, online intervention

## Abstract

**Background:**

Adults with conditions that affect their appearance, known as visible differences, can experience appearance concerns, social anxiety, and depression. Interventions have been developed for this population to facilitate adjustment and coping skills; however, they have limited evidence of efficacy. The Expand Your Horizon [Alleva, J. M., Martijn, C., Van Breukelen, G. J., Jansen, A., & Karos, K. (2015). Expand Your Horizon: A programme that improves body image and reduces self-objectification by training women to focus on body functionality. *Body Image,* 15, 81–89. https://doi.org/10.1016/j.bodyim.2015.07.001] online functionality-based writing programme was adapted for adults with visible differences.

**Method:**

A pilot randomised controlled trial with a wait-list control group was carried out to assess preliminary intervention efficacy and gain information about the acceptability and feasibility of the programme. Forty-four adults aged 21–63 years (*M* = 40.21; *SD* = 12.05) with visible differences took part. Various facets of body image (i.e. functionality appreciation and body appreciation) as well as depression and anxiety were assessed immediately pre- and post-intervention and at three-months.

**Results:**

Participants reported enjoying the programme, felt that the format was acceptable, and it significantly increased functionality appreciation, which was maintained at three-months. However, there were no improvements in body appreciation, depression, and anxiety.

**Conclusions:**

In future, a full trial should be carried out with an active control group.

## 1. Introduction

Health conditions that affect appearance, collectively known as ‘visible differences’, can have a negative impact on an individual’s quality of life and self-perception (Rumsey & Harcourt, 2012). Over one million people in the United Kingdom (UK) have a visible difference, which can be caused by a range of congenital conditions (e.g. cleft lip and/or palate, microtia) or acquired through injuries, diseases, or as a result of treatment (e.g. burn scarring, alopecia, mastectomy; Partridge & Julian, 2008). While visible differences vary in the physical challenges they present (e.g. pain, fatigue, itchiness, hearing or sight impairment, mobility issues), the psychosocial impact is comparable across conditions (Jenkinson et al., 2015; Rumsey & Harcourt, 2012). Although some adjust well to their condition, others may experience appearance dissatisfaction, body image concerns, appearance-related distress, low self-esteem, social anxiety, and depression, which can lead to social isolation and negatively impact life engagement (Norman & Moss, 2015; Rumsey & Harcourt, 2012). Having a visible difference may also impact an individual’s perception of their bodily self, which relates to the way an individual perceives, experiences, and interacts with their body, and how it shapes their sense of identity and the way they interact with the world (Sebri et al., 2021). Furthermore, much of this negative impact is derived from harmful representations of visible difference in the media (e.g. villains, victims), and experiences of appearance-related stigma and discrimination from members of the public (e.g. staring, unwanted questions, bullying; Stone & Wright, 2012; Thompson & Kent, 2001; Wardle et al., 2009).

Considering the potential negative impact, interventions have been developed to help individuals adjust to their condition and equip them with coping skills using techniques such as Cognitive Behavioural Therapy (CBT; Bessell et al., 2012; Clarke et al., 2013; Van Dalen et al., 2021), Social Skills Training (SST; Robinson et al., 1996), and Acceptance and Commitment Therapy (ACT; Powell et al., 2023; Zucchelli et al., 2021, 2022). Moreover, interventions including social support have been found to improve body image in women with appearance changes as a result of breast cancer and social support has been found to predict adjustment to a range of visible differences (Spatuzzi et al., 2016; Zucchelli et al., 2023). However, Norman and Moss (2015) systematic review identified that there is a lack of methodological rigour in current studies evaluating interventions for adults with visible differences, which makes it difficult to determine their efficacy. Therefore, it is necessary to carry out research using rigorous designs such as randomised controlled trials (RCTs). Moreover, Norman and Moss (2015) highlighted that it would be beneficial to develop more accessible interventions to provide support to more individuals.

Recently, researchers have considered the concept of *positive body image* in relation to adults with visible differences. Positive body image is associated with physical and psychosocial health and wellbeing and can be defined as ‘love and acceptance of one’s body (including aspects inconsistent with societally-prescribed ideals) and appreciation of its uniqueness and the functions it performs’ (Tiggemann, 2015). Theoretically, having a positive body image could protect individuals with visible differences from negative appearance-related messages from the media and members of the public, and encourage them to appreciate and embrace their body functionality (Harcourt & Williamson, 2019; Wood-Barcalow et al., 2010). Support for this comes from qualitative research, which finds adults who have successfully adjusted to a visible difference report focussing more on caring for their bodies than how they look and have come to appreciate and respect their bodies including what they can *do* (Egan et al., 2011; Garbett et al., 2017).

Moreover, a systematic review by Guest and colleagues (2019) identified that positive body image can be fostered in adult women through various interventions including a functionality-based writing programme, ‘Expand Your Horizon’. Out of the thirteen interventions included in the review, Expand Your Horizon had the most evidence of efficacy at improving positive body image. Specifically, the original intervention has evidence of efficacy with women aged 18–30 years, with effects maintained at one-month follow-up (Alleva et al., 2015; 2018a) and an adapted version of the programme increased body appreciation, functionality appreciation, and appearance satisfaction, and reduced depression in women with rheumatoid arthritis (Alleva et al., 2018b).

The programme is theoretically informed by Objectification Theory (Fredrickson & Roberts, 1997), which posits that through the way Western societies value women based on their appearance, women are socialised to self-objectify and base their worth on how they look. The programme challenges self-objectification by encouraging individuals to think about and appreciate their body functionality rather than appearance (Alleva et al., 2015). Individuals with visible differences are also susceptible to the same societal pressures which lead to self-objectification. Moreover, looking different from the ‘norm’ can lead individuals with visible differences to become hyper-aware of their appearance and experience high levels of social anxiety and fear of negative appearance evaluation, which can lead to anxiety, depression, and social isolation (Zucchelli et al., 2023). Therefore, the programme may be beneficial for adults with visible differences by encouraging them to think about what their body can do, rather than how it looks. Importantly, much of this work has to date been carried out with women-only samples; therefore, a recent systematic review highlighted the need for future research on positive body image interventions which include men (Guest et al., 2019).

Alleva and colleagues (2015) conceptualise body functionality holistically, as everything the body can *do* and not only relevant to able-bodied individuals (Alleva & Tylka, 2021). Body functionality has six facets: physical capabilities, internal bodily processes, creative endeavours, senses and sensations, communication, and self-care. Consistent with this, women with various visible, physical disabilities reported that functionality was an important aspect of their overall body image and that they focussed on their bodies’ internal processes and how their functionality had been adapted due to their conditions (Thomas et al., 2019). Additionally, support for the use of writing interventions with this population comes from Sherman and colleagues (2019), who found an online self-compassion-based writing intervention (My Changed Body) improved self-compassion and negative affect in adults with skin conditions. There are several theoretical mechanisms thought to underpin therapeutic and expressive writing. One which is particularly relevant to the Expand Your Horizon intervention is that writing and reflecting can enable an individual to better understand their experiences and the challenges they face, which can help to change their perspective on them (Pennebaker & Chung, 2007). In the case of the Expand Your Horizon intervention, participants are encouraged to shift their perspective on their body from how it looks to what it can do for them, thereby encouraging them to value functionality over appearance.

In summary, body image is a key concern for adults with visible differences; however, it is not the focus of most available interventions for this population (Rumsey & Harcourt, 2012). Additionally, to support a variety of individuals with visible differences, there is a need for interventions that are cost-effective, easily accessible, and self-directed (Norman & Moss, 2015). Research in the field of body image has identified that promoting positive body image may be more beneficial than addressing body dissatisfaction when it has already developed and is associated with increased physical and psychosocial wellbeing (Guest et al., 2021; Halliwell, 2015). One existing positive body image intervention, the Expand Your Horizon intervention, has evidence of improving positive body image, body dissatisfaction, and depression and is a self-directed (Guest et al., 2021). Taken together, these findings suggest that an online functionality-based writing intervention may be beneficial for adults with visible differences.

This pilot study aimed to investigate the preliminary efficacy of ‘Expand Your Horizon: More Than My Appearance’, an adapted version of the Expand Your Horizon intervention, with adults with a visible difference. The hypotheses were:

**Hypothesis 1:** After completing the Expand Your Horizon Intervention, there will be a statistically significant improvement in functionality appreciation scores in the intervention group compared to the control group, and this improvement will be maintained at the 3-month follow-up assessment.

**Hypothesis 2**: After completing the Expand Your Horizon Intervention, there will be a statistically significant improvement in body appreciation scores in the intervention group compared to the control group, and this improvement will be maintained at the 3-month follow-up assessment.

**Hypothesis 3:** After completing the Expand Your Horizon Intervention, there will be a statistically significant improvement in anxiety scores in the intervention group compared to the control group, and this improvement will be maintained at the 3-month follow-up assessment.

**Hypothesis 4:** After completing the Expand Your Horizon Intervention, there will be a statistically significant improvement in depression scores in the intervention group compared to the control group, and this improvement will be maintained at the 3-month follow-up assessment.

**Hypothesis 5:** Expand Your Horizon will be a feasible intervention for adults with visible differences.

**Hypothesis 6:** Expand Your Horizon will be an acceptable intervention to adults with visible differences.

## 2. Materials and methods

### 2.1 Participants and recruitment

**Figure 1.**
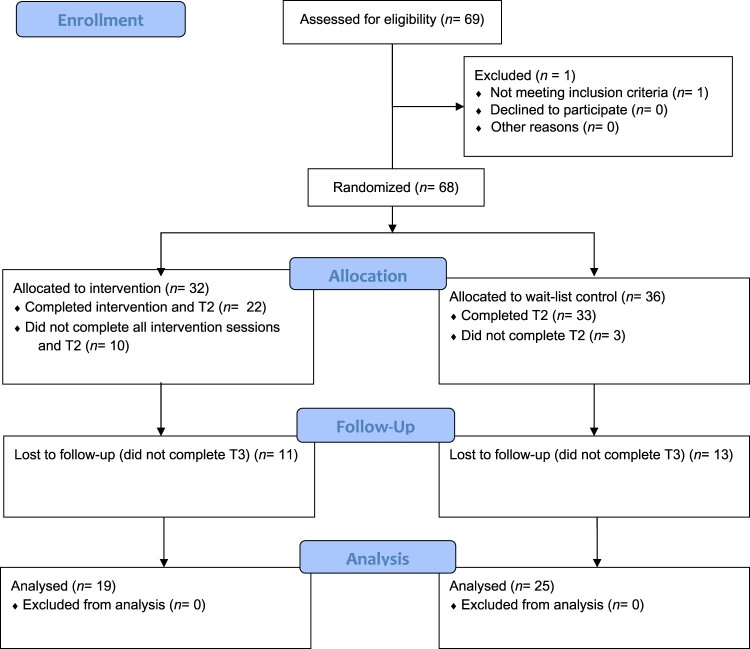
CONSORT (2012) Flow Diagram.

Sixty-eight adults with visible differences were enrolled into the study and completed the baseline measures (T1). After random allocation, fifty-five (80.88%) participants (intervention condition = 22; wait-list control = 33) completed the immediate-post measures (T2), and 44 (64.71%) participants (intervention = 19, control = 25) completed follow-up outcome measures at 3-months post-intervention (T3). Lower bound standardised effect sizes for a medium-sized effect and lower bound standardised effect for a large effect are commonly given at d = 0.5, and d = 0.8 respectively. A sensitivity power analysis (alpha = 0.05, two-sided) for a between-groups ANCOVA analysis indicates that for the achieved sample sizes the study would have 80% power for a standardised effect size (Cohen’s d) = 0.55 providing the correlation of measures between T1 and T2 is at least r = 0.8, and would have 80% power for a standardised effect size (Cohen’s d) = 0.63 for a correlation of r = 0.7. See Figure 1 for CONSORT flow diagram of participant allocation, Table 1 for participant characteristics, and Table 2 for visible difference information.

**Table 1.** Participant characteristics.

	Total Sample *n* (%)	Intervention Group *n* (%)	Control Group *n* (%)
**Gender**			
Male	12 (27.27%)	5 (26.32%)	7 (28%)
Female	30 (68.18%)	13 (68.42%)	17 (68%)
Other	2 (4.55%)	1 (5.26%)	1 (4%)
**Sexual Orientation**			
Heterosexual	37 (84.09%)	15 (78.95%)	22 (88%)
Homosexual	2 (4.55%)	2 (10.53%)	0 (0%)
Bisexual	2 (4.55%)	0 (0%)	1 (4%)
Other	1 (2.27%)	0 (0%)	0 (0%)
Prefer not to say	2 (4.44%)	2 (10.53%)	0 (0%)
**Marital status**			
Single	28 (63.64%)	9 (47.37%)	19 (76%)
Widowed	1 (2.27%)	0 (0%)	1 (4%)
Married/Civil partnered	14 (31.82%)	9 (47.37%)	5 (20%)
Prefer not to say	1 (2.27%)	1 (5.26%)	0 (0%)
**Ethnic Group**			
Asian	2 (4.55%)	1 (5.26%)	1 (4%)
Black	1 (2.27%)	1 (5.26%)	0 (0%)
Mixed/Multiple Ethnic Groups	3 (6.82%)	1 (5.26%)	2 (8%)
White	38 (86.36%)	16 (84.21%)	22 (88%)

**Table 2.** Information about visible differences of participants.

Visible Difference	Number reporting condition	Percentage of sample reporting condition
Cleft lip and/or palate	13	29.55%
Psoriasis	9	13.64%
Eczema	5	11.36%
Scarring	5	11.36%
Alopecia	2	4.55%
Birthmarks	2	4.55%
Rosacea	2	4.55%
Vitiligo	2	4.55%
Disorders of sex development	1	2.27%
Facial palsy	1	2.27%
Hydrocephalus	1	2.27%
Microtia	1	2.27%
Lipoedema	1	2.27%
Trichorhinophalangeal syndrome	1	2.27%
Strabismus	1	2.27%

Recruitment took place between January 2019 and December 2020. This included via a group of charities, known as the Appearance Collective, who work closely with the research centre carrying out the study. They sent information about the study to their members via email and their social media accounts. Additionally, a recruitment email was sent to members of a participant mailing list held by the centre, which contains the contact details of individuals who are interested in participating in the centre’s research. The study was also advertised via the Talk Health Partnership website and relevant Reddit communities (an online news and discussion forum with groups relating to different topics including visible differences). Individuals who were interested in the study contacted the researcher by telephone or email and those who chose to proceed were enrolled. To be eligible to take part, the participants had to be adults aged 18 years and above, be able to access the internet to complete the writing intervention and outcome measures, be English-speaking, and identify as having an appearance-altering condition or injury. Participants were excluded if they were under the age of 18 years, did not speak English, did not have an appearance-altering conditions, and could not access the online intervention and outcome measures. Dyslexia was not an exclusion criterion.

### 2.2 Measures

#### 2.2.1 Primary outcome measure

The primary outcome was functionality appreciation, measured immediately pre-intervention (T1), immediately post-intervention (T2) and at 3-month follow-up (T3). Those in the wait-list control condition completed the outcome measures at the same timepoints as those in the intervention condition and were then given the opportunity to complete Expand Your Horizon.

*Functionality Appreciation Scale (FAS;* Alleva et al., 2017***)*** measures how much someone appreciates their body functionality. The seven-item scale is scored on a five-point Likert scale from ‘strongly disagree’ to ‘strongly agree’, which are averaged to obtain a total score. Higher scores indicate greater functionality appreciation. Cronbach’s alpha scores for functionality appreciation were .94 at Time 1, .94 at Time 2, and .90 at Time 3. The FAS has evidence of internal consistency, construct validity, and test-retest reliability (Alleva et al., 2017).

#### 2.2.2 Secondary outcome measures

Body appreciation, anxiety, and depression were secondary outcomes, measured immediately pre-intervention (T1), immediately post-intervention (T2) and at 3-month follow-up (T3).

*Body Appreciation Scale-2 (**BAS-2;*** Tylka & Wood-Barcalow, 2015***)*** is a 10-item scale assessing body appreciation, scored on a five-point Likert scale from ‘never’ to ‘always’, with higher scores indicating greater body appreciation. A total score is derived by calculating the overall mean score. Cronbach’s alpha scores for body appreciation were .94 at Time 1, .93 at Time 2, and .95 at Time 3. The BAS-2 has evidence of internal consistency, construct validity, and test-retest reliability (Tylka & Wood-Barcalow, 2015).

*Patient-Reported Outcomes Measurement Information System (PROMIS)-Depression and Anxiety Short Forms* Each questionnaire consist of four items, scored on a five-point Likert scale from ‘never’ to ‘always’. Scores are averaged to obtain a total score, with higher scores reflecting greater symptoms of depression/anxiety. Both forms have evidence of psychometric validity and reliability (Pilkonis et al., 2011). Cronbach’s alphas for depression were .93 at Time 1, .92 at Time 2, and .89 at Time 3. For anxiety, they were .91 at Time 1, .90 at Time 2, and .90 at Time 3.

*Programme feedback* Information about the acceptability and feasibility of the Expand Your Horizon intervention was gathered retrospectively via a quantitative survey using five visual analogue scales (VAS) scored from 0 to 100. The questions assessed the mode of delivery (online format), affective attitudes towards the intervention (enjoyment, impact), and user experiences and perceptions in relation to the length of the intervention and number of writing exercises (Sekhon et al., 2017). Data were also collected relating to attrition. The data is presented in Table 4. The writing exercises were analysed for fidelity to assess whether the participants had adhered to the intervention writing tasks.

##### 2.2.2.1 Programme materials

The intervention was based on ‘Expand Your Horizon’ (Alleva et al., 2015), including its adaptation for women with rheumatoid arthritis (Alleva et al., 2018b). Intervention users are introduced to the six facets of body functionality (physical capabilities, internal processes, bodily senses, creative endeavours, communication, and self-care) and asked to undertake three online writing exercises over the course of five days. On Day 1, they are instructed to write about their bodily senses and physical capabilities, Day 3, their internal processes and creative endeavours, and Day 5, communication, and self-care. Beyond writing about their body functions, they are asked to reflect on why these functions are valuable to them. Examples are provided for writing inspiration, together with instructions to try and write for at least 15 min per exercise and take short breaks if needed.

The authors adapted the intervention to be gender neutral and specific to adults with visible differences. This included acknowledging that some people with appearance-altering conditions may also have functional mobility issues, but they should still focus on what their body *can* do, rather than what it *cannot*. The end of the intervention suggests other ways to think about body functionality and encourages users to come up with ideas to enhance their intentions. Feedback was gained on the programme materials from adults with visible differences, appearance psychology experts, and staff from organisations that provide support to individuals with visible differences at a training workshop and used to make final alterations. The intervention can be accessed here: Resources from the Centre for Appearance Research (CAR) - Appearance Research | UWE Bristol.

### 2.3 Procedure

The necessary ethics approvals were gained from the University of the West of England (UWE) Faculty Research Ethics Committee (HAS.16.12.072). Participants were enrolled onto the programme via SOTO (System for Online Training and Research), an online tool hosted by Maastricht University, which manages research studies. SOTO emailed participants the Information Sheet and Consent Form and, after enrolling, reminders to log-in and complete outcome measures and/or writing sessions. If participants did not complete a writing exercise within three days, SOTO removed them from the remainder of the study. The writing exercises and questionnaires were hosted on Qualtrics, an online survey software which allowed participant diary entries to be securely saved by the research team. SOTO randomised participants to the intervention or wait-list control using a 1:1 allocation ratio. Baseline measures (T1) were completed by both groups immediately after enrolment. The intervention group then completed the writing exercises on Days 1, 3, and 5. Post-intervention outcome measures were completed immediately after the third writing exercise (T2; Day 5) and at 3-month follow-up (T3). The control group completed outcome measures at 5 days (T2) and 3-months (T3) after enrolment and were then given access to the intervention. Participants were not aware of their allocated condition until after they had completed the pre-intervention (T1) outcome measures.

### 2.4 Data analysis

Analysis was carried out using Just Another Statistics Programme (JASP), an open-source statistics programme. A series of 2 (Group: intervention vs. wait-list control) x 2 (Time: T2 vs T3) mixed repeated-measures ANCOVAs were conducted to examine group differences across time for each outcome measure (functionality appreciation, body appreciation, anxiety, and depression), controlling for baseline (T1) scores. Little’s Missing Completely at Random (MCAR) Test was used to assess missing data at each timepoint. Outcome measures were checked for excessive skewness and kurtosis, deviations from normality, homogeneity of variances and covariances, and sphericity.

Qualitative content analysis was carried out to examine whether participants adhered to the writing tasks and to identify the contents of their diary entries. The purpose of qualitative content analysis is to understand the meaning of textual data by carrying out systematic coding to identify key categories and their frequency within the data (Forman & Damschroder, 2007). Deductive coding was used to analyse the writing task data in relation to the six areas of body functionality, which were used as a categorisation matrix. Data that did not relate to body functionality was coded inductively. The initial coding and data categorisation was carried out by the third author. Using the categories provided, the first author independently coded a random 20% of entries from each task as recommended by O’Connor and Joffe (2020). Where discrepancies were found, the intervention materials were referred to for final decision of coding category. Intercoder reliability for each code was calculated using Cohen’s Kappa and Landis and Koch’s (1975) guidance was used to interpret the Kappa values whereby 0–0.20 is ‘slight’ agreement, 0.21–0.40 is ‘fair’ agreement, 0.41–0.60 is ‘moderate’ agreement, 0.61–0.80 is ‘substantial’ agreement, and 0.81–1 is ‘nearly perfect’ agreement.

### 2.5. Ethics statement

(a) Institutional Review Board Statement: The study was conducted in accordance with the Declaration of Helsinki and was approved by an Institutional Review Board/Ethics committee. See details under Methods.

## 3. Results

### 3.1. Data preparation

Independent samples t-tests revealed no statistically significant baseline (T1) differences between the groups for functionality appreciation (*t*(66) = 0.95, *p* = 0.35), body appreciation (*t*(66) = 0.93, *p* = 0.35), depression (*t*(66) = 0.45, *p* = 0.65), or anxiety (*t*(66) = 0.42, *p* = 0.67). Chi-squared tests were carried out to examine differences in the categorical variables for the intervention and control groups. There were no significant associations for ethnicity (χ²(4) = 4.10, *p* = .39), age (χ²(36) = 42.60, *p* = .21), gender (χ²(2) = 0.15, *p* = .93) or marital status (χ²(4) = 5.91, *p* = .21). Little’s Missing Completely at Random (MCAR) Test was non-significant (*p* = 1.00), suggesting data were missing completely at random (Little, 1988). Missing data were 20.3% at T2 and 36.2% at T3. Intention to treat (ITT) analysis was carried out to assess preliminary efficacy (Jakobsen et al., 2017). Outcome measures were checked for excessive skewness and kurtosis, deviations from normality, homogeneity of variances and covariances, and sphericity and fell within acceptable ranges.

### 3.2 Intervention efficacy

Table 3 presents mean scores for the groups at each timepoint.

**Table 3.** Outcome measure means for each group at each time point.

	Intervention Group (*n* = 19)						Control Group (*n = 25)*							
	T1 Pre-Intervention (Baseline)		T2 Post- Intervention (5 Days Post Enrolment)		T3 3-Month Follow-Up		T1 Baseline		T2 5 Days Post – Enrolment		T3 3-Months Post – Enrolment			
Measure	*M*	*SD*	*M*	*SD*	*M*	*SD*	*M*	*SD*	*M*	*SD*	*M*	*SD*	F(1,41)	η2
Functionality appreciation	3.41	1.11	3.85*	0.79	3.82*	0.69	3.31	0.74	3.36	0.86	3.63	0.68	1.17	0.03
Body appreciation	2.70	0.87	2.93	0.73	2.92	0.94	2.64	0.70	2.66	0.75	2.77	0.73	0.25	0.01
Depression	2.33	1.17	2.21	1.14	2.34	1.00	2.87	1.02	2.69	1.06	2.75	0.90	0.16	0.00
Anxiety	2.40	1.16	2.51	1.13	2.53	1.15	2.72	1.09	2.58	1.05	2.58	0.84	0.18	0.00

*Indicates significant mean group differences.

#### 3.2.1 Primary outcome

##### 3.2.1.1 Functionality appreciation

For functionality appreciation, there was a significant main effect for Group, F(1, 41) = 4.53, *p* < .05, η2 = 0.1, indicating that, overall, participants in the intervention group reported higher functionality appreciation than participants in the control group. The difference in functionality appreciation between groups was the same at T2 as at T3, as demonstrated by the non-significant Group x Time interaction F(1, 41) = 1.17, *p* = .29, η2 = 0.03. That is, at both T2 and T3, participants in the intervention group reported higher functionality appreciation than participants in the control group. There was also a significant main effect of Time *F*(1,41) = 9.52, *p* < .005, η2 = 0.19, indicating that, overall, participants in both groups reported higher functionality appreciation at T3 than at T2.

#### 3.2.2 Secondary outcomes

##### 3.2.2.1 Body appreciation

For body appreciation, there was no significant main effect of Time *F*(1,41) = 2.54, *p* = .12, η2 = 0.06, or Group *F*(1,41) = 1.62, *p* = 0.21, η2 = 0.04 and no interaction effect *F*(1,41) = 0.25, *p* = 0.62, η2 = 0.01.

### 3.3. Depression

For depression, there was a significant main effect of Time *F*(1,41) = 8.38 *p* = .006, η2 = 0.17, with scores being higher at T3. The main effect of Group *F*(1,41) = 0.12 *p* = .74, η2 = 0.01 and the interaction effect *F*(1,41) = 0.16 *p* = 0.69, η2 = 0.00 were non-significant.

### 3.4. Anxiety

Similarly, for anxiety, there was a significant main effect of Time *F*(1,41) = 9.34 *p* <, 005, η2 = 0.19, with scores being higher at T3. The main effect of Group *F*(1,41) = 0.90 *p* = .35, η2 = 0.02 and the interaction effect *F*(1,41) = 0.18 *p* = .67, η2 = 0.00 were non-significant.

### 3.5. Intervention feedback

Overall, feedback from those in the intervention group was positive (see Table 4). Participants reported enjoying the intervention, that it had a positive impact on them, that it had made them think about their bodies more holistically and allowed them to be accepting and grateful for what their bodies could do, which enabled them to look beyond their visible difference and any physical limitations they had. Additionally, over 80% of participants completed the whole intervention. The number and length of the writing tasks were considered appropriate, and they liked the format. Qualtrics recorded the time spent on each of the writing tasks. Participants spent an average of 18.46 min (*SD* = 8.05) on Task One, 18 min (*SD* = 10.35) on Task Two and 17 min (*SD* = 7.26) on Task Three. Some commented that they would have liked the exercises to include thinking about their body functionality in relation to their specific visible difference in addition to body functionality generally.

**Table 4.** Acceptability data: Intervention Feedback.

Feedback Question	*M*	*SD*	Range of Scores
Overall, how much did you enjoy taking part in the programme? (0 = Not at all, 100 = Very much)	76.09	21.49	29–100
Overall, what kind of impact did the programme have on you? (0 = Very negative, 100 = Very positive)	71.26	19.72	38–100
What did you think about the number of writing exercises? (0 = Too few, 100 = Too many)	54.91	18.45	28–100
What did you think about the length of each writing exercise? (0 = Too short, 100 = Too long)	56.36	22.74	6–100
What did you think about the online format of the programme? (0 = Dislike, 100 = Like)	85.91	23.38	23–100

### 3.6. Content analysis of writing entries

Qualitative content analysis was carried out to examine the journal entries. Data for the six areas of body functionality was derived using deductive coding (physical abilities, senses and sensations, internal processes, creativity, self-care, communicating) and another six categories were developed inductively (appearance, daily life, physical health, mental health, my body, sense of self), which related to other topics written about in the journal entries. Cohen’s Kappa values calculated for each code to determine intercoder reliability. Intercoder reliability values for the deductive codes for the six areas of functionality were classified as ‘substantial’ (*n = 4*) to ‘almost perfect' (*n *= 2) agreement. The deductive codes relating to the other topics of the journal entries were classified as ‘moderate’ (*n = *3) to ‘substantial’ (*n =* 3) agreement. The Cohen’s Kappa values can be found in Table 5.

Participants wrote about all aspects of body functionality; however, descriptions relating to communication, self-care, and internal bodily processes were most prevalent. Participants also wrote about their appearance, daily life, physical and mental health, body connectedness, and their sense of self. Feeling connected with and appreciating their body, and aspects of mental health, were most written about. This suggests that the intervention had encouraged them to reflect beyond their specific body functionality and considered themselves and their bodies holistically. The findings suggest that it may be beneficial to assess whether the intervention improved positive embodiment, which is a broader concept than positive body image which refers to how individuals connect to and experience their bodies (Piran, 2002). Tables 5 and 6 present the writing categories and example excerpts from the participants’ entries.

**Table 5.** Content analysis of writing task data (Intervention group only).

Categories	Content written in writing tasks				Intercoder Reliability (Cohen’s Kappa)
	Task 1	Task 2	Task 3	Total	
**Areas of Body Functionality**					
1. Physical abilities (Task 1)	25.07%*	8.42%	3.05%	12.71%	0.67
2. Senses and sensations (Task 1)	12.06%*	2.63%	4.81%	6.75%	0.68
3. Internal processes (Task 2)	15.17%	21.71%*	10.92%	14.96%	0.70
4. Creativity (Task 2)	6.69%	18.29%*	4.11%	8.75%	0.83
5. Self-care (Task 3)	10.93%	14.08%	29.11%*	16.82%	0.71
6. Communicating (Task 3)	17.06%	8.16%	32.04%*	18.43%	0.87
**Other Categories**					
7. Appearance	7.54%	5.13%	6.46%	6.21%	0.64
8. Daily Life	5.66%	2.89%	3.05%	3.86%	0.50
9. Physical health	6.88%	9.08%	6.22%	6.96%	0.72
10. Mental health	10.93%	8.68%	12.79%	10.39%	0.63
11. Me and my body	13.76%	11.97%	5.63%	10.18%	0.59
12. Sense of self	5.94%	6.32%	4.58%	5.36%	0.59

*Notes: Participants were asked to explore this theme within the writing task. Number represents the count of that code relative to all other code count.

**Table 6.** Descriptions and examples of content analysis categories from participants’ writing entries.

Category	Description	Examples areas	Example Quotes
**Areas of Body Functionality**			
Physical abilities (Task One)	Physical things our bodies can do	Playing sports, flexibility, strength, coordination, stamina, balance, energy	*‘I am a keen gym goer’* ‘*Going out walking, yoga at home, exercising with music’* ‘*My body is capable of walking and venturing out’*
Senses and sensations (Task One)	Using our senses and experiencing our surroundings	Seeing, smelling, hearing, touching, tasting, enjoying and experiencing	*‘thankful for being able to smell beautiful perfumes and beautiful home cooked foods’.*
Internal processes (Task Two)	Processes that go on inside our bodies automatically	Using the brain (memory, imagination, learning), breathing, digestion, growth, healing, hormones	*‘[I use exercise] as a release, as a way of increasing my endorphins’* ‘*to hear inside my head the words I've used. I can type at the speed of my thoughts’*
Creativity (Task Two)	Using the body to engage in creative endeavours	Drawing, writing, gardening, playing games, cooking/baking	*‘I am quite adept at making cakes and quite creative at conjuring up a healthy meal with little planning and only scant regard for the recipe book’*
Self-care (Task Three)	Engaging in activities to take care of ourselves	Washing, relaxing, eating, drinking, comfort, rest, alone time	*‘I can go into the bathroom, wash my face, brush my teeth, comb my hair and moisturize my body’*
Relationships and communicating (Task Three)	Using the body to communicate and form/maintain relationships	Socialising, communicating, giving and receiving love, relationships, intimacy	*‘speak to my family and friends’* ‘*I like to chat with others … and enjoy their interaction’.*
**Other Categories**			
Appearance	How I and others perceive my looks	Confidence, self-image, my visible difference, others’ perception of my appearance	*‘[Relishing in how my body adapts] takes me away from focusing on my wobbly bit, or my skin condition’*
Daily life	My body allows me to live my life	Chores, routine, my job	*‘My body and brain allow me to work and be good at my job’* ‘*can focus, take charge, execute plans with results’*
Physical health		Health conditions, disability, injury and/or illness, treatments, pain	*‘Any way i am at the moment try to lose weight to get better health i am drinking more water more fruit and salads’*
Mental health		Mental health, emotions, mindfulness	*‘although anxiety often prevents [going for a walk]’*
Me and my body	My connection to my body	Feeling connected to body, pushing myself, learning about my body, gratitude and amazement towards the body	*‘relish in how my body adapts and transfers use to other things’*
Sense of self	What makes me ‘me’	Expressing myself, aging, spirituality/religion, being ‘normal’, my values	*‘[Playing tennis] is an activity that is really important to me as it acknowledges my sentience’*

## 4. Discussion

This pilot RCT has provided information about the feasibility, acceptability, and preliminary efficacy of the Expand Your Horizon programme for adults with visible differences. Those who completed the programme found it acceptable, adhered to the writing tasks, and demonstrated a holistic understanding of body functionality. Additionally, in relation to intervention efficacy, those in the intervention group experienced significantly higher functionality appreciation, both at post-test and at 3-month follow-up. On the other hand, the intervention did not have an impact on body appreciation, depression, or anxiety.

The improvements in functionality appreciation in those who completed Expand Your Horizon are in line with Alleva and colleagues’ (2018b) study, which found that functionality appreciation improved in women with rheumatoid arthritis who completed the programme. This is also consistent with the theoretical underpinnings of the intervention which aims to encourage individuals to focus on what their bodies can do, rather than how they look, and to reflect on what their bodies do for them (Alleva et al., 2015). The finding that functionality appreciation improved and was maintained at follow-up is promising because positive body image (which includes functionality appreciation) is related to improved overall physical and psychosocial health and wellbeing (Linardon et al., 2023). This includes carrying out behaviours relating to physical health such as sleep hygiene, adaptive eating, and attending medical screenings (Andrew et al., 2016; Gillen, 2015). Furthermore, positive body image is related to improved psychosocial wellbeing including self-esteem, self-compassion, optimism, and life satisfaction (Halliwell, 2015; Tylka & Wood-Barcalow, 2015). Therefore, there is potential for the intervention to have broader positive implications in relation to overall wellbeing.

Conversely, the finding that body appreciation did not significantly increase after completing the intervention contradicts the findings of other studies examining the effectiveness of Expand Your Horizon (i.e. Alleva et al., 2015; 2018b). Notably, however, in the original trial, Alleva and colleagues (2015) found that the improvements in body appreciation were only marginally significant. Body appreciation is a component of positive body image which includes appreciating both the function *and* appearance of the body (Tiggemann, 2015). Therefore, it is possible that while the intervention did encourage the participants to appreciate what their body does for them, it may not have actively encouraged them to appreciate, or change their perceptions about, their appearance. Indeed, the three writing exercises do not focus on appreciating aspects of appearance. However, the participants did write about feeling connected with their bodies, which suggests that it may be beneficial to measure embodiment in future trials. Embodiment is a broader concept, related to positive body image, which conceptualises how individuals connect with and experience their bodies (Piran, 2002).

The intervention also had no significant impact on levels of anxiety or depression. In Alleva and colleagues’ (2018b) evaluation of Expand Your Horizon with women with rheumatoid arthritis, the intervention led to decreases in depression but not anxiety. The authors suggest that the strength-based nature of the intervention may be more suited to promoting wellbeing than alleviating anxiety (Alleva et al., 2018b). Nonetheless, in contrast with the findings of this study, Alleva and colleagues did find significant reductions in depression in their sample. A possible explanation for the findings in the current study is that depression and anxiety, which are commonplace in individuals with visible differences (Rumsey & Harcourt, 2012; van Dalen et al., 2020), can often be attributed to negative experiences with others, such as receiving unwanted attention (e.g. comments, questions, staring) due to having a condition that is noticeable (Stone & Wright, 2012; Thompson & Kent, 2001; Wardle et al., 2009). Consequently, Expand Your Horizon, which focusses on changing one’s perceptions of their own body functionality, may therefore not have targeted the main causes of anxiety and depression in adults with visible differences. There are existing visible difference interventions that decrease depression and anxiety in this population by developing coping strategies (e.g. Clarke et al., 2013; Zucchelli et al., 2022); however, these do not target body image, which is also a key concern for individuals with visible differences. Therefore, having a toolbox of different interventions may provide the best approach for supporting this population. Moreover, as many of the concerns adults with visible differences face are a result of experiences with others, it is also important to target the stigmatising attitudes and behaviours of society, rather than putting the onus on an individual to cope with negative experiences and discrimination (Mathews et al., 2023).

It is promising that the intervention effects were maintained at 3-month follow-up, suggesting the intervention changed the participants’ way of thinking about their body functionality for at least several months. However, to fully determine efficacy it is necessary to assess whether improvements are maintained in the longer-term and whether top-up sessions are needed to sustain these effects.

Another consideration is that the sample included both men and women. Guest and colleagues’ (2021) systematic review identified a lack of effective positive body image interventions for men. However, the findings from this study suggest Expand Your Horizon may be beneficial for them. Future research could usefully explore the efficacy of Expand Your Horizon with men from the general population.

The feedback from participants who completed the whole programme suggested that they liked the format (online writing tasks) and felt that the length and number of tasks was appropriate. However, there were relatively high attrition levels and feedback was not gained from those who dropped out, meaning it is likely to be skewed towards those who enjoyed it and potential participation barriers are unclear. Although online interventions have the benefits of being widely accessible and cost-effective, attrition rates are often high due to the time commitment, volume of materials, and use of technology (Sherman et al., 2019). While over 80 percent of participants completed the whole intervention and post-intervention outcome measures, under 65 percent completed the 3-month follow-up measure, which included the feedback questions. However, web-based intervention follow-up rates have been found to be as low as 11% in some research (Khadjesari et al., 2011). One way to improve attrition in future may be to provide incentives to participants for completing each timepoint rather than using a prize draw (Khadjesari et al., 2011).

Within the current study, it was not possible to explore participant burden; therefore, it would also be beneficial to gain feedback from those who dropped out of the study to explore the barriers to completing the online intervention to make it more accessible to all adults with visible differences. Additionally, there may be other functionality-based intervention techniques that would be easier for some individuals to engage with (e.g. thinking about body functionality, writing short statements about functionality). The findings suggest the online writing-based format may be acceptable to some individuals, but not all, and highlights the need for a variety of approaches to suit individual preferences (Harcourt et al., 2018). Another technique to encourage functionality appreciation that has been suggested by researchers in the field is reflecting on body functionality whilst carrying out physical activities, which may be more suitable for individuals who do not enjoy reflective writing (Alleva et al., 2020).

The content analysis enabled the authors to examine the writing entries in detail. Adults with visible differences did engage with writing about and reflecting on all aspects of body functionality. Interestingly, communication, self-care, and internal bodily processes were most written about. This shows that they have been able to engage with and appreciate the more nuanced and abstract aspects of body functionality, rather than just those that relate to physical abilities.

Although instructed to focus on what their bodies *can* do, many of the participants began by focussing on their body’s limitations, particularly relating to those caused by their appearance-altering condition. However, they then moved on to focussing on, and appreciating, what their bodies can do. It seems that it may be important for individuals completing the intervention to acknowledge their functional limitations in order to then move on from these and embrace other aspects of body functionality. Therefore, it could be useful to include this information in the intervention instructions and to explain that it can take time to focus on body functionality in a positive way. Furthermore, each writing exercise contained various examples of functions, some of which may have highlighted a function affected by a specific appearance-altering condition (e.g. hearing, smiling, running). It is unclear what impact, if any, this may have had on participants’ writing experiences. As appearance-altering conditions and injuries vary widely, it may be beneficial to assess which examples are most appropriate for interventions which are not condition-specific. Additionally, it is necessary to ensure a wide variety of examples are included so that individuals completing the intervention can identify functions that relate to their personal experiences, even if their condition impacts some aspects of their body functionality.

The participants also reflected on their mental health, the connection they had to their bodies, and their sense of self, which relates to body and functionality appreciation. These findings could help to tailor the instructions to people with visible differences, such as including specific examples identified in the content analysis. In line with Objectification Theory (Fredrickson & Roberts, 1997), which underpins the programme, participants had successfully shifted their focus from evaluating their worth based on their appearance to focussing on their functionality and valuing other aspects of themselves. Moreover, in line with theories relating to reflective writing, the participants had been able to change their perspective on their experiences and the potential challenges they face through the writing exercises (Pennebaker & Chung, 2007).

### 4.1 Methodological considerations

There are various strengths of the design, including the use of an online RCT, the three-month follow-up assessment, and the intervention being completed as it would in a real-world setting. Furthermore, collecting the writing task entries anonymously allowed the authors to identify which aspects of functionality were relevant to the group; however, it is possible that collecting the writing data may have impacted how honest the participants felt that they could be with the writing tasks, which may reduce its ecological validity.

The authors used various avenues to expand recruitment to a wide range of individuals with visible differences; however, most participants were White. Therefore, additional avenues, including recruiting via specific community groups and social media pages for racial and ethnic minority groups may help to overcome this issue in future. In relation to assessing acceptability and feasibility, data were gathered relating to the mode of delivery, contents of the intervention, number of writing exercises, affective attitudes towards the intervention, user experience and satisfaction (Sekhon et al., 2017). However, adherence and participant burden in relation to those who dropped out of the intervention were not collected. Therefore, it would be beneficial to gain feedback on the reasons for this in future trials. Finally, while this study has provided initial information on the feasibility, acceptability, and preliminary efficacy of the intervention for individuals with a visible difference, a full trial with an active control group and larger sample is needed to fully determine efficacy.

### 4.2 Practical implications

The findings of this study provide numerous practical implications. First, as a self-directed intervention that can be accessed online, Expand Your Horizon can be and used by adults with visible differences at no cost. Additionally, it is possible to download a PDF version of the resource and use it offline, which may be beneficial for individuals who do not regularly use the internet. It has also provided a resource that charitable organisations supporting adults with visible differences can include on their webpages and signpost their members to. Furthermore, it may be used alongside other therapeutic techniques such as CBT and ACT within services that provide one-to-one psychological support to adults with visible differences, such as the Outlook Service in the UK (Kleve et al., 2002).

### 4.3 Conclusions

This pilot study provides preliminary evidence that the adapted version of Expand Your Horizon, an online functionality-based writing intervention, may be an acceptable and cost-effective, freely accessible intervention, for adults with visible differences, which improves functionality appreciation up to 3-month follow-up. However, a full RCT should be carried out in future to fully determine its efficacy.

## Data Availability

Anonymous data can be made available on request.
